# Frequency doubling by active *in vivo* motility of mechanosensory neurons in the mosquito ear

**DOI:** 10.1098/rsos.171082

**Published:** 2018-01-10

**Authors:** James F. C. Windmill, Joseph C. Jackson, Victoria G. Pook, Daniel Robert

**Affiliations:** School of Biological Sciences, University of Bristol, Bristol Life Sciences Building, 24 Tyndall Ave, Bristol BS8 1TH, UK

**Keywords:** nanomechanics, neurons, motility, atomic force microscopy, mosquito

## Abstract

Across vertebrate and invertebrate species, nonlinear active mechanisms are employed to increase the sensitivity and acuity of hearing. In mosquitoes, the antennal hearing organs are known to use active force feedback to enhance auditory acuity to female generated sounds. This sophisticated form of signal processing involves active nonlinear events that are proposed to rely on the motile properties of mechanoreceptor neurons. The fundamental physical mechanism for active auditory mechanics is theorized to rely on a synchronization of motile neurons, with a characteristic frequency doubling of the force generated by an ensemble of motile mechanoreceptors. There is however no direct biomechanical evidence at the mechanoreceptor level, hindering further understanding of the fundamental mechanisms of sensitive hearing. Here, using *in situ* and *in vivo* atomic force microscopy, we measure and characterize the mechanical response of mechanosensory neuron units during forced oscillations of the hearing organ. Mechanoreceptor responses exhibit the hallmark of nonlinear feedback for force generation, with movements at twice the stimulus frequency, associated with auditory amplification. Simultaneous electrophysiological recordings exhibit similar response features, notably a frequency doubling of the firing rate. This evidence points to the nature of the mechanism, whereby active hearing in mosquitoes emerges from the double-frequency response of the auditory neurons. These results open up the opportunity to directly investigate active cellular mechanics in auditory systems, and they also reveal a pathway to study the nanoscale biomechanics and its dynamics of cells beyond the sense of hearing.

## 1. Introduction

**Figure 1.**
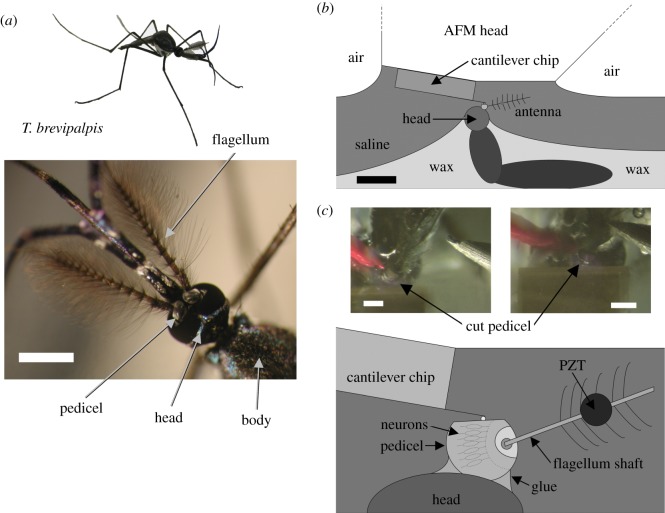
Nanoscale *in vivo* and *in situ* force microscopy. (*a*) Male *Toxorhynchites brevipalpis* with close-up of head and plumose antennae. Scale bar, 1 mm. (*b*) Overall schematic of the preparation. Scale bar, 3 mm. (*c*) Close up schematic of the preparation. The PZT stimulus position is shown by the circle. Two photos of the preparation under the AFM are shown in the inset: left shows the preparation before the AFM's approach to the cut pedicel, right shows the AFM cantilever in position for measurements. Scale bars, 300 µm.

The key proximal function of auditory systems is to transform sound into mechanical vibrations that, in turn, are transduced into nervous impulses by specialized mechanosensory cells—neurons in the case of insects. In the ears of mosquitoes, transduction occurs in mechanically sensitive ciliated neurons, located within Johnston's organ in the second segment of the insect's antenna (the pedicel, figure 1*a*). Johnston's organ contains a surprisingly large number of mechanoreceptors, about 16 000 [1], a number equivalent to that of sensory epithelial hair cells in the human ear. Each mechanoreceptor unit, known as a scolopidium, is a multi-cellular cluster that essentially comprises: (i) one or several mechanosensory neurons each endowed with a central sensory cilium, (ii) a scolopale cell that envelops the neurons and contains a series of stiff scolopale rods, and (iii) attachment cells at the distal end linking the neuron and scolopale cells to the external auditory anatomy [2]. This basic layout varies substantially between different species [1–3], the exact functions associated with the respective cellular scolopidial elements, and their structural characteristics, being not clearly understood.

For all auditory systems, a major limitation to our understanding of the mechanisms of active auditory amplification has been the lack of direct mechanical data taken *in situ* from mechanoreceptors *in vivo*. Here we present such data, revealing the *in situ* nanometre-scale mechanical response of grouped mechanoreceptor units *in vivo* under ecologically and physiologically relevant conditions.

In the mosquito species *Toxorhynchites brevipalpis* the male's Johnston's organ has been shown to produce neural signals in response to deflections of the tip of the antenna of just ±7 nm [4]. Considering that the length of the antennal flagellum is *ca* 3.3 mm in this species, the angular deflection detected at threshold by the mechanosensitive neurons is estimated at about ±0.0001° [4]. This equates to sub-nanometre displacement sensitivity in the auditory neurons at the base of the antenna. Such sensitivity is typical of insect auditory systems, exemplified by the moth tympanal ear, where neural signals were measured in response to mechanical displacements of just 100 pm [5]. In moth ears, the mechanoreceptor cell is directly connected to the vibrating tympanal membrane, whereas in the mosquito a lever system couples the antennal flagellum to the several thousand mechanoreceptors in Johnston's organ [6]. Both mechanical and electrophysiological evidence therefore suggests that the mosquito's mechanosensory cells are sensitive to sub-nanometre displacements, yet measurements at the cellular level have remained elusive.

The mosquito antennal hearing apparatus is of interest because of its active auditory mechanics. This is a process by which the overall mechanical activity of the auditory system is enhanced, conferring nonlinear sensitivity and frequency selectivity [7]. This active process shows striking similarities to that of cochlear amplification, whereby the auditory hair cells of vertebrates actively amplify the sound-induced vibrations to which they respond [8–11]. Therefore, the mosquito ear is considered a suitable and effective model system for active hearing [12]. In brief, the antennal response of *T. brevipalpis* displays active nonlinear features involving amplification, compression, and hysteresis. Hypotheses on the cellular motile mechanisms required to achieve this have been proposed but not demonstrated [13,14]. A mathematical model has been developed that is capable of describing the known features of nonlinear dynamics in *T. brevipalpis* [15], a model that postulates that an ensemble of mechanosensory neurons, behaving as nonlinear oscillators, is entrained to the antennal motion once the stimulus level is above a certain threshold. The model predicts that this is only achievable if the ensemble of neurons (as nonlinear oscillators) are driven at twice their natural frequency. In this case, once the stimulus level crosses a threshold, a pitchfork bifurcation occurs and the neurons entrain their natural oscillation to the stimulus. In the human ear, active auditory mechanics is the result of motility in the outer hair cells within the cochlea [9,15], derived from the expression of the mechanical work produced by the hair cell. Motility has been attributed to a change in the length of the outer hair cells, generated by a lattice of the prestin protein [16] and/or changes in the overall mechanical compliance of the cell's hair bundle through adaptation [11]. This way, by producing mechanical work coherent (i.e. phase-locked) with mechanical inputs, outer hair cell motion can reinforce, through regulated phasic feedback, the amplified and frequency-selective basilar membrane motions. Active auditory mechanics is phylogenetically widespread; motility of mechanosensory cells and its contributions to acute hearing is also reported to take place in turtles [17] and frogs [18,19] as well as mammals. In insects, as in the mosquito *T. brevipalpis*, and in the fruit fly *Drosophila*, active mechanisms are considered to rely on the mechanical action of the ciliated mechanoreceptors [20–23]. Enticingly, a prestin homologue is deemed to be present in the mechanosensory cells of Johnston's organ; however, this was shown not to contribute to the motile process in *Drosophila* [24,25]. Active auditory mechanisms have also been demonstrated in cricket species, presenting a tympanal ear rather than the antennal Johnston's organ based receiver, establishing a yet broader phylogenetic spread of the mechanism [26].

Studying the complex dynamics of active auditory mechanisms presents many challenges, not least due to the limited access to the mechanoreceptor cells within a living animal. Although it is technically possible to produce images of cells in a living preparation, or in a whole animal, direct mechanical measurements of motile auditory cells have necessitated the isolation of the cells, or the dissection of the tissue holding them. This has been done to great effect in several studies, for example using atomic force microscopy on isolated guinea pig outer hair cells [27] or glass fibres in contact with hair cells from the sacculus of the bullfrog [18]. Such studies thus miss the full contextual information of how these cells interact with the rest of the system, as they would *in situ* and *in vivo*. It has remained a major challenge to undertake meaningful mechanical measurements of the motion and forces that mechanosensory cells undergo when they are in their natural, *in situ*, functional dynamic state. That capability would open further insight into how mechanosensory systems function as a whole, with appropriate measurements of their constituent parts. The work reported here takes steps toward solving this problem, which while not the perfect answer, presents a preparation and *in situ* and *in vivo* mechanical responses from a mechanosensory auditory system.

## 2. Material and methods

### 2.1. Animals

Experiments were performed using 25 adult male *T. brevipalpis* mosquitoes (figure 1*a*). Mosquito eggs were obtained from the London School of Tropical Hygiene and Medicine (UK). During all stages, the predatory larvae were fed both liver powder and *Aedes aegypti* larvae ad libitum. Adult mosquitoes were kept at 25–27°C and 70% relative humidity, and fed with sugar water (5%) ad libitum. All experiments were performed at room temperature 23–25°C. Animals were anaesthetized with CO_2_, and mounted in low-melting-point paraffin wax (Agar Scientific Ltd, Stansted, UK) ventral-side up with their flagella pointing in a posterior direction. This allowed direct access to the animal's Johnston organ from above. The pedicel was then glued in position (Octal cyanoacrylate, World Precision Instruments Ltd, Stevenage, UK). After being submerged in saline, a small cut was made in the uppermost cuticular surface of the pedicel using a vibrotome (DTK-1500E DSK Microslicer, Dosaka EM, Kyoto, Japan). The preparation was kept submerged in saline for the duration of the experiment (figure 1*b*,*c*). The saline used in these experiments contained (in mM) 223 NaCl, 6.8 KCl, 8 CaCl_2_, 5.1 MgCl_2_, and 10 N-2-hydroxyethylpiperazine-N-2-ethanesulfonic acid, pH 7.8 [28].

### 2.2. Atomic force microscopy measurements

Mechanoreceptor unit vibrations were examined in the time domain in response to piezoelectric stimulation of the mosquito antenna with single frequency sinusoidal signals in the range 200–600 Hz. The vibrations were analysed by simultaneous recording of the vibration displacement of the mechanoreceptor units, and the displacement of a closed loop piezoelectric (PZT) actuator (P841.10 Physik Instrumente, Karlsruhe, Germany), excited by supplying a voltage to the actuator's modular control system (E-500.00/E-509.S3/E-505.00 Physik Instrumente). The displacement of the closed loop piezoelectric actuator was calibrated with sub-nanometre accuracy using a microscanning laser Doppler vibrometer (Polytec PSV-300-F, Waldbronn, Germany) with an OFV-056 scanning head. The displacement of the piezoelectric actuator and external antenna was confirmed to be the single stimulus frequency. Calibration was regularly verified between experiments to ensure no change had occurred. Cellular displacement was measured using an atomic force microscope (AFM; JPK Nanowizard, Berlin, Germany), mounted on a vibration isolation table (TMC 63-500 series, Technical Manufacturing Corp., Peabody, MA, USA). Measurements were carried out in saline, in contact mode, using standard cantilevers (DNP silicon nitride cantilevers, Veeco, USA) with 10 µm diameter spherical polystyrene beads attached at the end of the cantilever [29]. Therefore, the AFM probe is in contact with 1 or more scolopale units, in addition to local support cells, as it is not possible to locate its proximity to the boundary between units. The cantilever sensitivity and stiffness were calibrated using the AFM's own built-in functions. As the AFM used is closed-loop in all three axes the cantilever vertical sensitivity calibration is highly accurate (vertical noise less than 80 pm). The cantilever stiffness (spring constant) calibration was also carried out using the thermal noise method [30]. The AFM cantilever position was monitored via a live video feed (DFK 31F03 Firewire Color CCD camera: 1024 × 768 pixels, Imaging Source, Bremen, Germany; MZ7-5 Zoom Stereomicroscope, Achromat 1.0× objective, 89 mm working distance, 1.0× video-/photo tube A, Leica Microsystems, Wetzlar, Germany) to the AFM's controlling computer (figure 1*c*). The AFM's approach routine was used to bring the cantilever into contact with the mechanoreceptor units. During the experiment the AFM cantilever is stationary (not scanning), but the AFM height control feedback is left running to ensure the cantilever signal does not drift. This very slow feedback was seen not to affect the higher frequency stimulation and measurement. Measurements could be taken at any point on the preparation by repositioning the head of the AFM.

### 2.3. Extracellular electrophysiology

Neural activity was measured as compound action potentials (CAPs) from mechanoreceptor neurons in Johnston's organ recorded extracellularly via an electrolytically sharpened tungsten electrode inserted directly into the pedicel. Commercially available electrodes could not be used as the pedicel's external cuticle is too hard for them to penetrate. Customized electrodes, made by hand, were created to overcome this problem. For grounding, a second electrode was inserted into the mosquito's abdomen. CAPs were recorded from a subset of the pedicel's mechanoreceptor neurons, and not the antennal nerve. CAP preamplification was carried out using a custom-made amplifier [31].

### 2.4. Signal processing and control

The AFM cantilever displacement signal, piezoelectric feedback signal, and amplified CAP voltage signal were simultaneously digitally sampled using a USB data acquisition system (CompactDAQ, National Instruments, Austin, Texas, USA), connected to a secondary computer system. The AFM cantilever signal, the PZT feedback voltage, and the CAP voltage signal were monitored and controlled by custom software written in the LabVIEW software environment (National Instruments, Austin, Texas, USA). This arrangement allows the measured voltage signals, and controlling PZT voltage output, to be updated simultaneously. The analysis of the cantilever vibration, neural activity, and the PZT feedback was carried out off-line using a set of custom written LabVIEW programs.

### 2.5. Modelling

It is sufficient to only consider a spatially extended form of the complex Ginzburg--Landau equation (CGLE) with 2 : 1 forcing from [15], where *A* *=* *A*(*x*, *t*) can be considered the force generated by the oscillators, Δ is the Laplace operator, and *α*_*i*_ are complex numbers such that

2.1 $$\frac{\delta A}{\delta t}=\alpha_{1}A+\alpha_{2}|A{|}^{2}A+\alpha_{3}A^{*}+\alpha_{4}\Delta A.$$

Here, *α*_3_ is the drive parameter, and as the AFM experiments directly force the antenna, it suffices to change this parameter, rather than have a coupled harmonic oscillator equation governing this parameter. In this sense we are removing the passive oscillator from the system, and are only therefore requiring the CGLE. The characteristic frequency of the oscillators, i.e. the frequency of oscillation when the system undergoes a Hopf bifurcation, is the rotating frame of reference of this equation. The imaginary part of *α*_1_ models the distance of the stimulus frequency to twice the characteristic frequency of the nonlinear oscillators. To solve this, the equation was discretized into 200 coupled equations (the Laplace operator replaced by *A_i+_*_1_ − 2*A_i_* *+* *A_i_*_−1_) representing 200 scolopidia. The initial conditions were uniformly randomly distributed. The drive parameter (a triangle ramp in time) was spatially weighted such that the force tended to zero at the spatial boundaries. Parameters used were *α*_1_ = *μ *− 0.16*i*, *α*_2_ = 0.57* *− 1.6*i*, *α*_3_ = 8.75*f*(*t*), *α*_4_ = 3, where *f*(*t*) is the triangle ramp from 0 to 1. In figure 4, for *μ* > 0, *μ* = 2, and for *μ* < 0, *μ* = −3.45.

## 3. Results

An operative procedure was developed to gain access to the mosquito Johnston's organ's mechanosensory units, while maintaining viability of the specimen. The cuticular shell of the pedicel is very hard relative to its muscular connection to the head capsule, necessitating a careful surgical approach. Cutting an adequate access window into the pedicel is critical and needs to preserve the functionality and physiological integrity of the auditory system. Accessing the preparation with an AFM cantilever required high positional accuracy, stability and repeatability in the surgical procedure. To perform such nanoscale mechanical measurements *in vivo*, the entire set-up was located on a vibration isolation table within an acoustically isolated booth. In particular, the mechanical stimulation provided by the PZT actuator to the antennal shaft was designed to prevent any crosstalk with the auditory organ and the AFM probe (figure 1; Material and methods).

**Figure 2.**
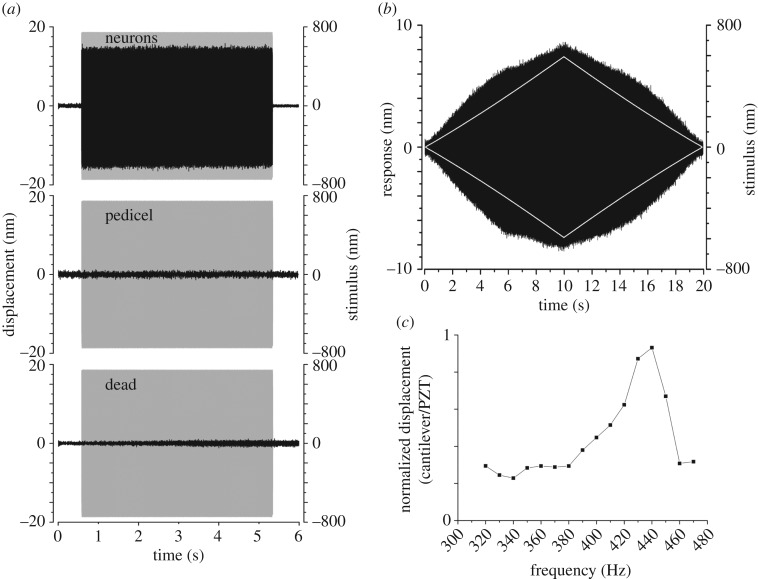
Atomic force microscopy on mechanosensory units. (*a*) The mechanical response of the mechanoreceptor units, the pedicel, and the mechanoreceptors of a freshly killed mosquito (black graphs) to a single frequency stimulus of 400 Hz, ±740 nm delivered at antenna (grey envelope). (*b*) Mechanical response (black) to a linear amplitude modulated tone stimulus (white, amplitude envelope), 400 Hz, ±600 nm. (*c*) Normalized mechanical responses to AFM measurements over a range of single stimulus frequencies.

The shaft of the antennal sound receiver was actuated at a sinusoidal frequency of 400 Hz with ±740 nm amplitude (figure 2*a*). This actuation generated a motion of ±15 nm, measured with the tip of the AFM probe located at the mechanoreceptor units (figure 2*a*). The AFM probe tip is in contact with 1 or 2 scolopale cells, in addition to local support cells, depending on its proximity to the boundary between cells. The displacement ratio of cellular response to actuation was 0.02 (15/740), i.e. 2%. Across individual mosquitoes, the average response at 400 Hz in these conditions was 2.15 ± 0.79% (mean ± s.d.; *N* = 25). Geometrically, the AFM cantilever can be regarded as being positioned on or about the scolopale rods, measuring motion perpendicularly to the longitudinal axis of the scolopale. Therefore, as the antennal flagellum is set into motion, the mechanoreceptor cilium, scolopale rods and envelope cells are stretched and compressed. The AFM measures motion due to this stretch--compression cycle, which is relevant to mechanoelectrical transduction. Yet, other parts of the auditory system could also be moving, a possibility that needs assessing.

To assess stimulus specificity and adequacy, the measurement was repeated with the AFM cantilever resting on the cuticular surface of the pedicel, next to the opening made to access the mechanoreceptor units. Actuation of the antennal shaft did not cause motion of the pedicelar cuticle (figure 2*a*). Notably, noise levels on the pedicel cuticle during actuation were 2.1 nm peak to peak (0.74 nm RMS). Additionally, when the cantilever was retracted 7.5 µm from the surface of the cells, but still bathed in the surrounding saline, vibrations were strongly reduced, not exceeding 0.9 nm peak to peak (0.32 nm RMS). Finally, mechanical actuation of the mechanoreceptor units of a freshly dead mosquito was assessed using the same stimulus. Here, the measured vibration level was 2.4 nm peak to peak (0.85 nm RMS). This control measurement was systematically carried out on the same mosquito that displayed mechanical and neural responses while alive. Altogether, this evidence shows that *in situ in vivo* measurement of mechanoreceptor unit, and surrounding support cell, vibrations is feasible and reliable; the PZT controlled mechanical actuation of the antennal shaft can specifically deliver mechanical energy to the mechanoreceptor units and not to the entire preparation. These measurements were also carried out at 350 and 450 Hz, yielding similar results to those at 400 Hz.

One important test of auditory nonlinearity characterizes the system's response to amplitude modulated mechanical actuation. All mosquitoes examined here were thus subjected to an amplitude-modulated stimulus with a pure-tone spectral content (as in [12]) (figure 2*b*). Remarkably, the mosquito mechanoreceptor units responded nonlinearly, much like an intact antennal flagellum to amplitude-modulated acoustic forcing [12]. Here, a nonlinear response is seen as the amplitude of the 400 Hz stimulus increases (figure 2*b*). The mechanical response of the units to mechanical stimulation of the flagellum was measured across a range of frequencies (figure 2*c*). This spectral response shows that the mechanical response of the mechanoreceptor units, as measured by the AFM cantilever, lies within the range of the mechanical response of the antennal flagellum [4,7]. In both mechanical cases, a maximal response magnitude was observed at frequencies around 430–440 Hz. Thus, the experimental preparation of the mosquito's auditory system, including the partial removal of the pedicel cuticular shell surrounding the mechanosensory organ, produces responses to stimuli equivalent to those of a whole specimen.

**Figure 3.**
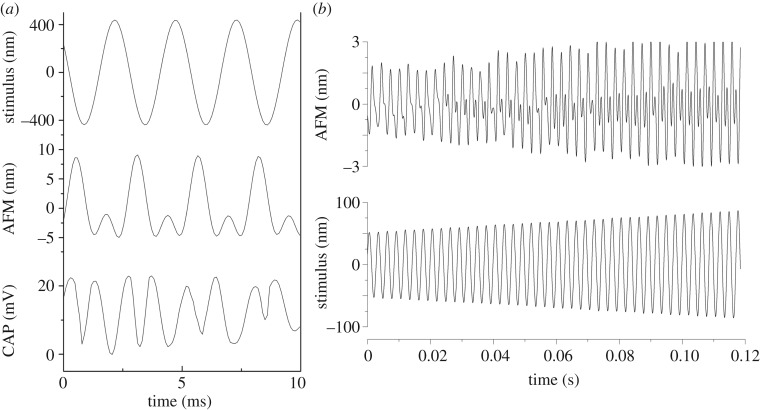
Nonlinear mechanical responses of mechanosensory units. (*a*) Simultaneous mechanical responses from mechanoreceptor units, and compound electrical responses of a subset of mechanoreceptor neurons, to a tone stimulus of 400 Hz show a second (doubled) frequency component. (*b*) Mechanical response of mechanoreceptor units to an amplitude-modulated stimulus with a pure-tone spectral content (350 Hz) reveals the emergence of a second (doubled) frequency component with increasing stimulus.

Previous experiments indicate that the compound rate of neuron firing within Johnston's organ is double that of the sound stimulus [6]. This intriguing result was established to be a localized effect, independent from the radial symmetry of the mechanoreceptor cells around the antenna [6]. Analysis of the mechanical response of the mechanoreceptor cells to tonal amplitude modulation clearly demonstrates that the mechanoreceptors are also moving with a second (doubled) frequency component (figure 3). Simultaneous extracellular CAP recordings reveal a subset of neurons responding with a frequency doubling component that is in line with the mechanical response (figure 3).

**Figure 4.**
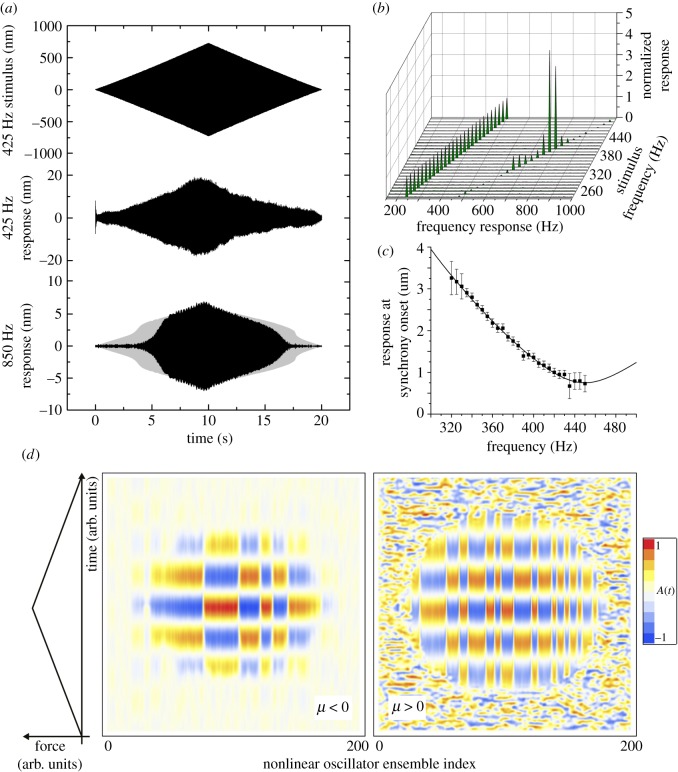
Mechanical twice-frequency response of the mechanosensory units. (*a*) The mechanical response of the mechanoreceptor units, at the stimulus frequency and twice the stimulus frequency (nominal antennal nonlinear response in grey), to a linear amplitude-modulated tone stimulus of 425 Hz, ±700 nm. (*b*) The mechanical frequency response of the mechanoreceptor units across a number of stimulus frequencies, showing that the second frequency only appears over a tight range of frequencies, which match the fundamental flight tone of the female. Data are shown for a single insect. (*c*) The external antennal response measured using laser vibrometry, indicating the association between response nonlinearity and stimulus frequency. For frequencies greater than 425 Hz (±20.7 Hz) no amplification occurs (*n* = 7). Solid line from the model described in [15]. (*d*) Pattern formation in an array of nonlinear oscillators. For sufficiently large forces, the array of oscillators, modelled as in [15], exhibit spatial biphasic patterns. The temporal oscillation is slowed down for illustration purposes—in reality the temporal oscillation has a period much shorter than the time scale of the stimulus. The plots represent two cases—a quiescent array (*μ* < 0) and an active array (*μ* > 0) that shows noisy fluctuations with no spatial patterning. Regardless, a strong force is sufficient and necessary to elicit local patterning, which is therefore a feature of the model of [15] and supports the hypothesis that Johnston's organ is entrained in a 2 : 1 resonance.

Similarly to the mechanosensory electrical response [15], frequency doubling of mechanoreceptor units' motion only occurs when the mosquito auditory system exhibits active mechanical amplification (figure 4*a*), betrayed by the antenna's nonlinear mechanical response to sound [12]. Mechanical amplification depends on stimulus frequency, and is only observed within the range 300–430 Hz in oscillating antennae [12]. Correspondingly, the mechanical measurement at the site of the mechanosensory units is in the frequency range of biological relevance to the insect (figure 4*b*).

The cessation of the frequency doubling response at approximately 430 Hz and above prompts a functional explanation. A mathematical model has previously described the nonlinear mechanics of the mosquito antenna as deriving from the synchronization of force generation in an ensemble of neurons [15]. In general terms, if the sound played at the antenna is *f* Hz, then the net force generation from neurons is mathematically predicted to occur at 2*f* Hz, in order to replicate well the overall nonlinear response of the antenna. Note that the model makes no prediction on where the frequency doubling happens between flagellum and neurons, just that it is necessary. Through model-based calculations and laser Doppler vibrometry data, an upper limit for this effect was predicted to occur at 425 ± 20.7 Hz (figure 4*c*, see [32]), at which point the model predicts that neurons can no longer maintain a twice-frequency entrainment, possibly due to the inherent refractory period of the typical mechanosensory neurons being too long. The AFM data presented match this prediction very closely, thus demonstrating not only the presence of frequency doubling in the motion of mechanoreceptor unit groups, but also matching theoretical predictions on the upper frequency limit of this effect.

## 4. Discussion

The contact measurement of the mechanoreceptor unit tissue in *T. brevipalpis* using an AFM shows a direct mechanical link between antennal and mechanoreceptor unit vibrations. The presence of a strong frequency doubled component in an otherwise undistorted signal reinforces the hypothesis that, rather than a by-product of some unknown mechanical property, this effect is strongly correlated with the active neuromechanical amplification produced by the Johnston's organ's neurons. The emergence of a frequency-doubled signal in the nanoscale displacements of a localized small population of neurons is coincident with the stimulus amplitudes that introduce the step-wise (switch-like) transitions seen in the antennal displacement [12,15], adding weight to the hypothesis that a twice-frequency force-generation response is the basis for the nonlinear dynamics of mosquito audition. A phase shift can be seen to occur between the mechanical and electrical response measurements (figure 3). This could relate in part to the geometric layout of the antenna and mechanoreceptor units, such that depending on which side of the antenna the mechanoreceptor unit vibrations were recorded there may be a switch in the phase of motion. Furthermore, the AFM measures the mechanoreceptor unit stretch--compression cycle. Therefore, it could be possible that the phase shift is due to how the stretch--compression appears in the AFM signal in comparison to the piezoelectric drive that moves the antenna back and forth.

These AFM experiments add weight to the twice-frequency hypothesis, in keeping with a previous model for mosquito hearing [15]. Here, antennal stimulation was achieved with an actuation rod far stiffer than the antenna (unlike in the sound-driven stimulation experiment) and as such the model presented in [15] can be simplified to consist of a non-resonant CGLE representing the force generation from a nonlinear oscillator ensemble, without the effect of the coupled antennal oscillator. A spatially extended CGLE model can be posited that describes the force from a linear array of coupled nonlinear oscillators, or in other terms, a nonlinear oscillatory medium. This model is capable of generating patterns of biphasic responses to a twice-frequency (or 2 : 1) stimulus [33,34] regardless of whether this ensemble is self-oscillatory or not. Figure 4*d* shows the simulation of a one-dimensional CGLE subject to 2 : 1 forcing, for both a self-oscillating, noisy array (*μ* > 0) and a quiescent array (*μ* < 0), using parameter values similar to ones used in full simulations of the mosquito antenna [15].

Biphasic spatial patterns can be elicited as the driving force increases (temporal oscillation is shown on a time scale to allow the reader to see the oscillation—in reality the temporal oscillation is far too fast to view graphically). Given that this model mimics the antennal nonlinear response to sound [12,15], it is possible the AFM is measuring local biphasic patterns in the ensemble of scolopidia, induced by the presence of a sufficiently strong twice frequency driving force, and that this biphasic response is possibly a necessary feature of active amplification in the mosquito ear. A biphasic response would be unusual in that it would avoid a limitation of a phasic neuron not able to respond to a too fast oscillatory stimulus. A correlated pair of neurons, as an effective single unit, would be able to respond to this stimulus by responding in turn. The stability of this distributed phasic response would be important to maintaining the stability of the antennal response—that the model shows the generation of stable spatial patterns induced by the force indicates that the mosquito ear can stably overcome the possible limitation in the scolopidia. A final point is that the onset of pattern formation, the biphasic response, and the antennal amplification events are effectively a switch dependent on stimulus—this switch can potentially be a behavioural cue for the mosquito during its courtship/pursuit.

We speculate therefore that the dynamics of sound detection, mechanically, are as follows. An acoustic stimulus of frequency *f* drives the antenna into motion, at frequency *f*. At the base, via some unknown mechanism, the neurons which attach to this are driven at a frequency 2*f*. Despite this, each neuron can only generate force at frequencies similar to the *f*. Once a sufficient intensity is reached, the ensemble of neurons entrain to the 2*f* drive but spatially extended, such that force generation is biphasic and once summed appears at 2*f*. This feedback reinforces the antennal motion via the previously stated unknown mechanism, thus amplifying the signal. It is easy to speculate further that the unknown mechanism is simply the result of a nonlinear transfer function between flagellum motion and neurons, where the 2 : 1 harmonic is present but, with sufficient intensity, is reinforced by neural force generation. This could explain the twice-frequency behaviour in figure 4*a*. This is an exciting avenue for future work to uncover the complex nature of signal detection in hearing systems.

## 5. Conclusion

The work presented here constitutes the first evidence of mechanical response and nonlinear activity in auditory mechanosensory units. *In situ* and *in vivo* mechanical events take place at magnitudes of nanometres and below, a scale that is biophysically and physiologically relevant in mosquito hearing. Control measurements provide evidence of the absence of mechanical crosstalk; the cellular mechanical response is in effect not accompanied by vibrations of other tissue. This result is important since it shows that the technique used succeeds at stimulating the delicate auditory machinery with specificity and adequate amplitudes. Remarkably, the ratio between the vibration amplitude of the antennal shaft, at biologically relevant amplitudes [12] and the mechanosensory unit tissue mechanical response is *ca* 2%. Using an AFM set-up dedicated to biological systems, the monitoring of mechanical signals with such a large difference in scale is feasible, offering direct access to the process of mechanoreception at the length scale of cells and molecules. Future work could combine the AFM approach with live imaging to provide further information of the process of mechanoreception at the cellular level. Enticingly, this approach could also be applied *in situ* and *in vivo* to other cell types, tissues, and physiological processes beyond hearing.

## Data accessibility

Data for piezoelectric stimulus (nanometres), neuron motion (nanometres) and compound action potentials (voltage) are openly available from the University of Strathclyde Pure/KnowledgeBase (http://dx.doi.org/10.15129/112924ac-d39a-4ab2-aa6b-2f078a5df864) [35].
